# Regulation of Embden–Meyerhof–Parnas (EMP) Pathway and Tricarboxylic Acid (TCA) Cycle Concerning Aberrant Chilling Injury Behavior in Postharvest Papaya (*Carica papaya* L.)

**DOI:** 10.3390/ijms241813898

**Published:** 2023-09-09

**Authors:** Lijin Huang, Shoukui Tao, Yi Zhu, Yonggui Pan, Zhengke Zhang, Zhiqian Yu, Yezhen Chen

**Affiliations:** 1 School of Food Science and Engineering, Hainan University, Haikou 570228, China; huanglijin339@163.com (L.H.); taoshoukui@126.com (S.T.); zhujiawen151@163.com (Y.Z.); zhangzhengke@hainanu.edu.cn (Z.Z.); yzhiqian2022@163.com (Z.Y.); chenyz29900@163.com (Y.C.); 2 Key Laboratory of Food Nutrition and Functional Food of Hainan Province, Haikou 570228, China

**Keywords:** papaya, chilling injury, respiration pathway, EMP pathway, TCA cycle

## Abstract

Postharvest abnormal chilling injury (CI) behavior in papaya (*Carica papaya* L.) fruit is a rare phenomenon that may be associated with respiratory metabolism. This study thus aimed to investigate the impacts of storage temperatures (1 and 6 °C) on the respiratory metabolism of postharvest papaya and its impact on CI development. Results demonstrated that 1 °C storage reduced the activities of hexokinase (HK), phosphofructokinase (PFK), pyruvate kinase (PK), citrate synthase (CS), and α-ketoglutarate dehydrogenase (α-KGDH) and regulated the expression of corresponding enzymes in the Embden–Meyerhof–Parnas (EMP) pathway and tricarboxylic acid (TCA) cycle compared with 6 °C storage, resulting in a lower respiration rate of the EMP-TCA pathway and mitigating the development of CI. Meanwhile, lower contents of nicotinamide adenine dinucleotide (hydrogen) (NAD(H)) were observed in papaya fruit stored at 1 °C. Notably, papaya fruit stored at 1 °C maintained higher activity and transcriptional levels of SDH and IDH during the whole storage period. These findings suggest that 1 °C storage reduced the respiration rate of the EMP-TCA pathway by reducing the expression level and activity of related enzymes, which is conducive to the reduction of respiration substrate consumption and finally alleviating the occurrence of CI.

## 1. Introduction

Papaya (*Carica papaya* L.) originates from Central and South America but is cultivated in all tropical regions [1]. The total world papaya area has reached 468,731 hectares and global production of papaya is estimated to rise by 2.1 percent each year, up to 16.6 million tons in 2029 [2,3]. But papaya is a tropical fruit that is prone to physical damage and disease, which poses challenges for its storage and transportation [4,5]. Among various preservation strategies, low-temperature storage has shown great potential and effectiveness in extending the storage life of papaya fruit [6]. However, papaya is climacteric fruit that is susceptible to chilling injury (CI) during cold storage [7]. Chilling injury (CI) is a physiological defect of plants following exposure to low but nonfreezing temperatures [8]. Notably, papaya fruit exhibits higher tolerance to CI when stored at 1 °C than 6 °C, which is a unique phenomenon and rarely observed in other cold-sensitive fruit [6]. This abnormal CI behavior in papaya fruit has attracted attention but its potential mechanisms remain unclear. Therefore, it is crucial to investigate the mechanism behind this abnormal CI behavior to enhance the resistance of papaya fruit to low-temperature stress.

Papaya is a climacteric fruit whose respiration rate increases significantly after harvest, and its respiration rate is affected by storage temperature [9,10]. Respiration is a crucial metabolic process in postharvest horticultural crops, which involves several pathways including the Embden–Meyerhof–Parnas (EMP) pathway, tricarboxylic acid (TCA) cycle, phosphopentose pathway (PPP), and electron transport pathway (ETP) [11,12]. The EMP-TCA pathway is a fundamental respiratory pathway that begins with the cytoplasmic oxidation of glucose to pyruvate and is followed by pyruvate entering mitochondrial reactions [13]. PPP is an alternative respiratory pathway that supplies reduced nicotinamide adenine dinucleotide phosphate (NADPH), which is a crucial reducing agent and intermediate of PPP [14,15]. Under normal environmental conditions, EMP-TCA is the main respiration pathway in plants. However, the respiratory pathway and intensity in fruit and vegetables can be influenced by external environmental stresses and various treatments [16]. Numerous studies have indicated that enhancing the respiratory metabolism of the PPP pathway can improve chilling tolerance in horticultural products [17,18,19]. For instance, treatment with nitric oxide (NO) alleviated the CI symptom in peach fruit and increased its antioxidant capacity by stimulating the PPP pathway and suppressing the EMP pathway [18]. Previous research has shown that the improved chilling endurance in papaya stored at 1 °C is related to the up-regulation of key genes (*CpG6PDH* and *Cp6PGDH*) in the PPP pathway [20]. However, there is no existing report on the changes in the EMP-TCA respiratory pathways and their relationship with abnormal CI behavior in papaya during cold storage.

The EMP-TCA pathway is the primary respiratory pathway responsible for the utilization of substances to generate energy and other compounds [13]. It has been proposed that reduced resistance of horticultural crops to stress is closely associated with high respiration rates and low energy status [21,22,23]. For example, a higher proportion of respiration of the EMP pathway, TCA cycle, and cytochrome pathway (CCP) in longans fruit treated with hydrogen peroxide (H_2_O_2_) resulted in increased consumption of metabolic substrates, leading to accelerated damage to the pulp structure and shortened shelf life [22]. Tan et al. [23] reported that the melatonin treatment of Chinese cabbage decreased the proportion of the EMP pathway, TCA cycle, and CCP while promoting the proportion of the PPP pathway. This adjustment inhibited excessive material consumption, ensured sufficient energy supply, and ultimately delayed senescence.

Respiratory metabolism is a complex physiological process regulated by the enzymatic activities of multiple respiratory pathways [24]. Hexokinase (HK), phosphofructokinase (PFK), and pyruvate kinase (PK) are the key enzymes in the EMP pathway that play important roles in regulating respiration rate [25,26]. The TCA cycle involves rate-limiting reactions catalyzed by citrate synthase (CS), isocitrate dehydrogenase (IDH), α-ketoglutarate dehydrogenase (α-KGDH), and succinate dehydrogenase (SDH) [16,27]. Under various biotic or abiotic stresses, the activities of enzymes involved in metabolism can be altered in different respiration pathways, thereby modulating the respiration rate and enhancing plant stress resistance [16]. H_2_S treatment effectively attenuated the progression of the CI by suppressing the activities of HK, PFK, PK, phosphor-glucose isomerase (PGI), and the levels of glucose and pyruvate, among other components of the EMP-TCA pathway [28]. Moreover, nectarine fruit at 0 °C demonstrated superior chilling tolerance compared to those stored at 5 °C, which was closely associated with the increased activity of SDH [29]. 

Therefore, the present study aimed to investigate the variations in the EMP-TCA pathway in papaya fruit subjected to 1 and 6 °C during storage. Specifically, we analyzed the levels of nicotinamide adenine dinucleotide (NAD) and reduced nicotinamide adenine dinucleotide (NADH), as well as the activities of metabolism-related enzymes and gene expressions involved in the EMP-TCA pathway. 

## 2. Results 

### 2.1. CI Index Development

**Figure 1:**
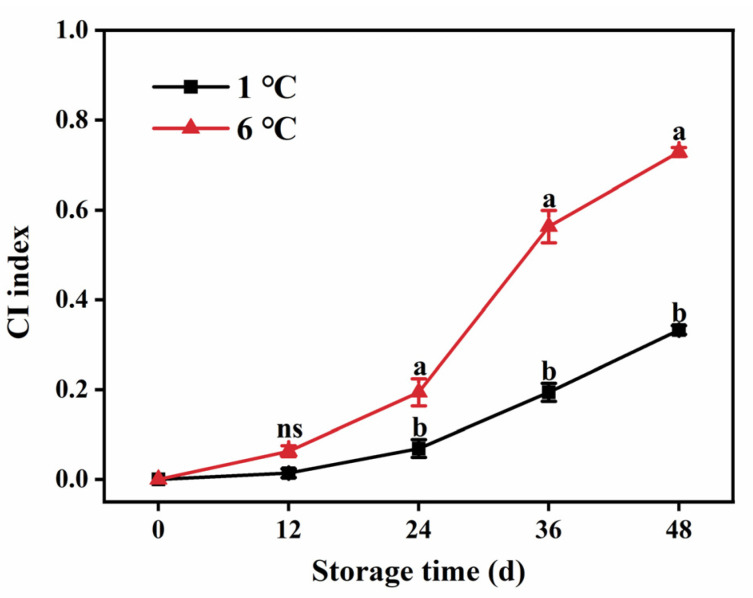
CI index development of papaya fruit stored at 1 and 6 °C during storage. Vertical bars represent the standard deviation of the means from three measurements. Different lowercase letters indicate significant differences (*p* < 0.05) between the 1 and 6 °C treatments in the same storage period. ns = Not significant. (■) 1 °C, (▲) 6 °C.

The CI index serves as a critical indicator of the severity of CI in papaya fruit [14]. In papaya fruit stored at 6 °C, the CI index became apparent after day 12 and progressively escalated with the extension of the storage period (Figure 1). However, when stored at 1 °C, the development of the CI index was significantly inhibited. On the final day of storage, the CI index of papaya fruit stored at 1 °C was 54.28% lower than that of papaya stored at 6 °C (Figure 1).

### 2.2. Respiration Rate

**Figure 2:**
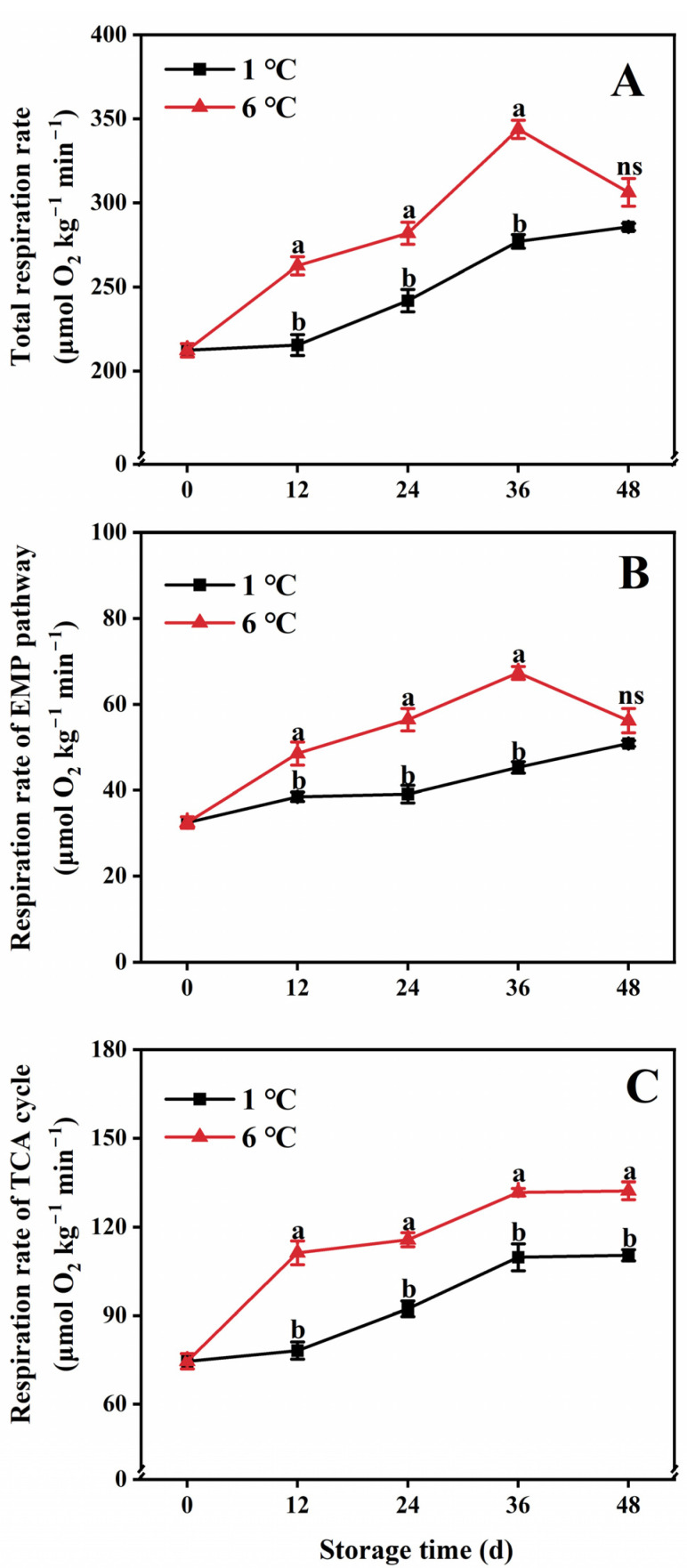
Total respiration rate (**A**) and respiration rate of the EMP pathway (**B**) and TCA cycle (**C**) in papaya fruit during storage at 1 and 6 °C. Vertical bars represent the standard deviation of the means from three measurements. Different lowercase letters indicate significant differences (*p* < 0.05) between the 1 and 6 °C treatments in the same storage period. ns = Not significant. (■) 1 °C, (▲) 6 °C.

The changes in both the total respiration rate and respiration rate of the EMP pathway and TCA cycle exhibited similar patterns in papaya fruit throughout the entire storage period at 1 and 6 °C (Figure 2). Initially, there was an upward trend, followed by a gradual decline at 6 °C. The peak total respiration rate occurred on the 36th day, reaching 343.58 ± 5.40 μmol O_2_ kg^−1^ min^−1^. In contrast, at 1 °C, the total respiration rate showed a gradual increase, but it was lower than that at 6 °C during the whole storage period (Figure 2A). Moreover, storage at 1 °C led to a reduction in the respiration rate of both the TCA cycle and the EMP pathway compared to storage at 6 °C, maintaining them at lower levels during the entire storage duration (Figure 2B,C).

### 2.3. NAD(H) Contents

**Figure 3:**
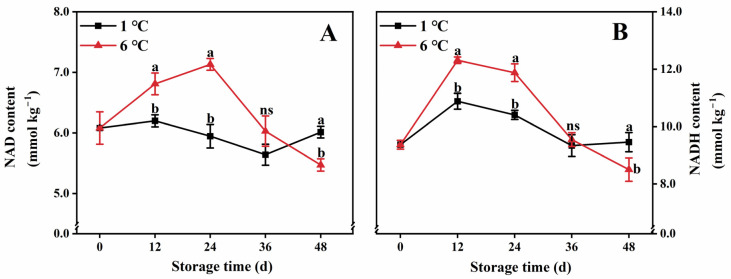
NAD (**A**) and NADH content (**B**) in papaya fruit during storage at 1 and 6 °C. Vertical bars represent the standard deviation of the means from three measurements. Different lowercase letters indicate significant differences (*p* < 0.05) between the 1 and 6 °C treatments in the same storage period. ns = Not significant. (■) 1 °C, (▲) 6 °C.

The changes in both NAD and NADH contents in papaya held at 1 °C and 6 °C displayed a similar pattern (Figure 3A,B). In papaya fruit stored at 6 °C, the NAD content increased within the first 24 d and subsequently experienced a gradual decline from days 24 to 48 (Figure 3A). On the other hand, compared to papaya fruit stored at 6 °C, the fruit stored at 1 °C exhibited lower NAD content for most of the storage period, specifically from days 12 to 36 (Figure 3A). Regarding NADH content, both 1 °C- and 6 °C-stored papaya fruit showed an initial increase during the first 12 days, followed by a steady decline (Figure 3B). Throughout the storage duration, 1 °C-stored fruit exhibited lower NADH content than 6 °C-stored fruit except at day 48 (Figure 3B).

### 2.4. Key Enzyme Activities in the EMP Pathway

**Figure 4:**
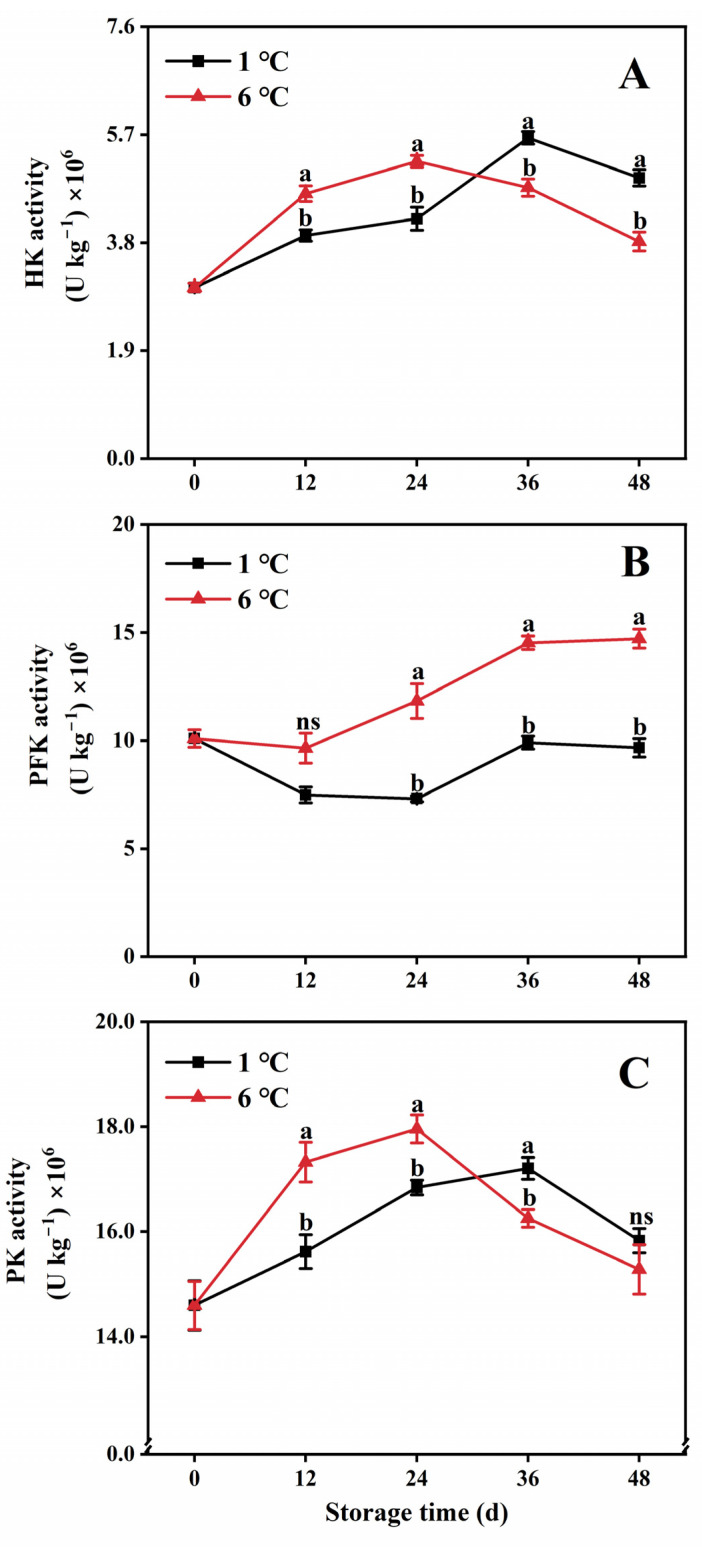
HK activity (**A**), PFK activity (**B**), and PK activity (**C**) in papaya fruit during storage at 1 and 6 °C. Vertical bars represent the standard deviation of the means from three measurements. Different lowercase letters indicate significant differences (*p* < 0.05) between the 1 and 6 °C treatments in the same storage period. ns = Not significant. (■) 1 °C, (▲) 6 °C.

In this study, papaya fruit stored at 1 °C and 6 °C initially exhibited an increase in HK and PK activities during the first 36 and 24 days, respectively, which then gradually declined (Figure 4A,C). Notably, the activities of HK and PK of fruit held at 1 °C were significantly suppressed during the first 24 days compared to fruit stored at 6 °C. However, after 36 days, HK activity rebounded and became higher, more than 1.18 times higher than that of fruit refrigerated at 6 °C (Figure 4A,C).

Furthermore, storage at 1 °C inhibited the activity of PFK throughout the entire storage period, with reductions of 38.2%, 31.8%, and 34.2% on days 24, 36, and 48, respectively, compared to storage at 6 °C (Figure 4B).

### 2.5. Key Enzyme Activity in the TCA Cycle

**Figure 5:**
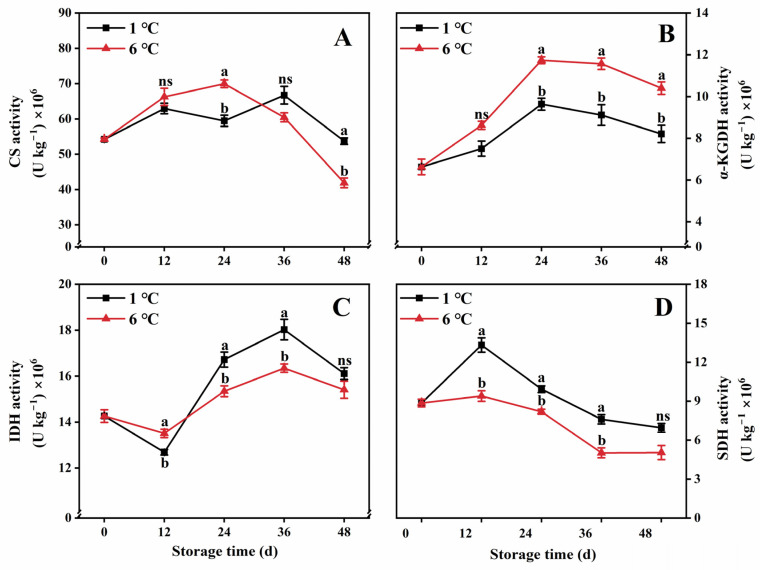
CS activity (**A**), α-KGDH activity (**B**), IDH activity (**C**), and SDH activity (**D**) in papaya fruit during storage at 1 and 6 °C. Vertical bars represent the standard deviation of the means from three measurements. Different lowercase letters indicate significant differences (*p* < 0.05) between the 1 and 6 °C treatments in the same storage period. ns = Not significant. (■) 1 °C, (▲) 6 °C.

Both CS and α-KGDH activities in 6 °C-stored fruit continuously increased and reached their peak at 24 d, followed by a decline for the remainder of the storage period (Figure 5A,B). In contrast, fruit stored at 1 °C exhibited lower CS activity from 12 to 24 d and lower α-KGDH activity from 12 to 48 d during storage, in comparison to fruit stored at 6 °C (Figure 5A,B).

IDH activity in 6 °C-stored fruit mildly declined during the first 12 d of storage, followed by a sharp increase from 12 to 36 d, and then a gradual decline until the end of the storage period (Figure 5C). On the other hand, fruit stored at 1°C demonstrated improved IDH activity, with statistically higher levels observed in 1°C-stored fruit compared to 6 °C-stored fruit during most of the storage duration (Figure 5C). Regarding SDH activity, 6 °C-stored fruit showed a slight rise within the initial 12 d of storage, followed by a subsequent decrease (Figure 5D). However, 1°C-stored fruit exhibited higher SDH activity than 6 °C-stored fruit throughout the entire storage period (Figure 5D).

### 2.6. Gene Expression

#### 2.6.1. Expression of EMP Genes

**Figure 6:**
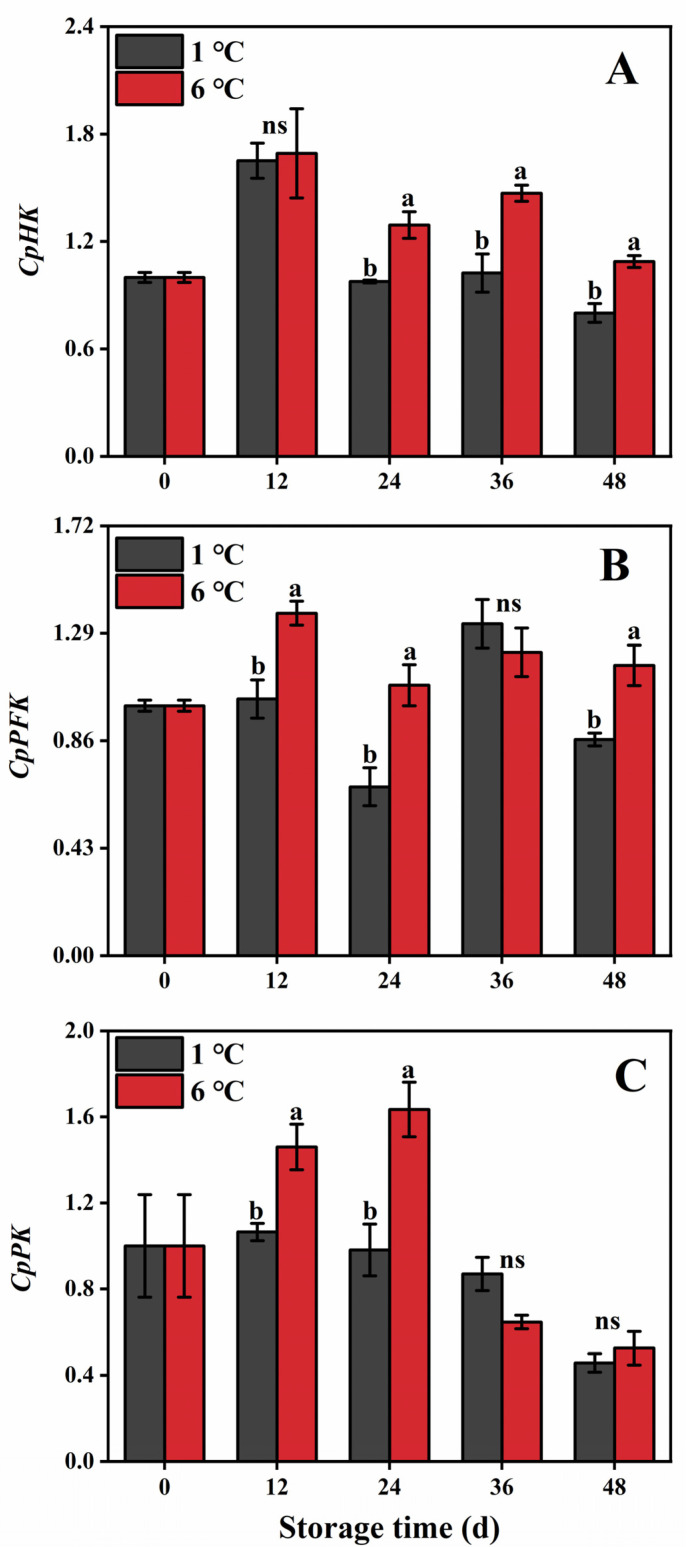
*CpHK* (**A**), *CpPFK* (**B**), and *CpPK* (**C**) genes’ expression in papaya fruit during storage at 1 and 6 °C. Vertical bars represent the standard deviation of the means from three measurements. Different lowercase letters indicate significant differences (*p* < 0.05) between the 1 and 6 °C treatments in the same storage period. ns = Not significant.

The expression of *CpHK* was found to be higher in fruit stores at 6 °C, particularly at 24, 36, and 48 d (Figure 6A). Conversely, during the same storage period, fruit stored at 1 °C exhibited suppression of the relative expression level of *CpHK* (Figure 6A). In 6 °C-stored fruit, the expression of *CpPFK* and *CpPK* was initially up-regulated and then down-regulated, with the peaks of *CpPFK* and *CpPK* occurring on 12 and 24 d, respectively (Figure 6B,C). However, 1 °C-stored fruit down-regulated the expression of these genes compared to 6 °C-stored fruit, except on day 36 (Figure 6B,C).

#### 2.6.2. Expression of Genes in TCA

**Figure 7:**
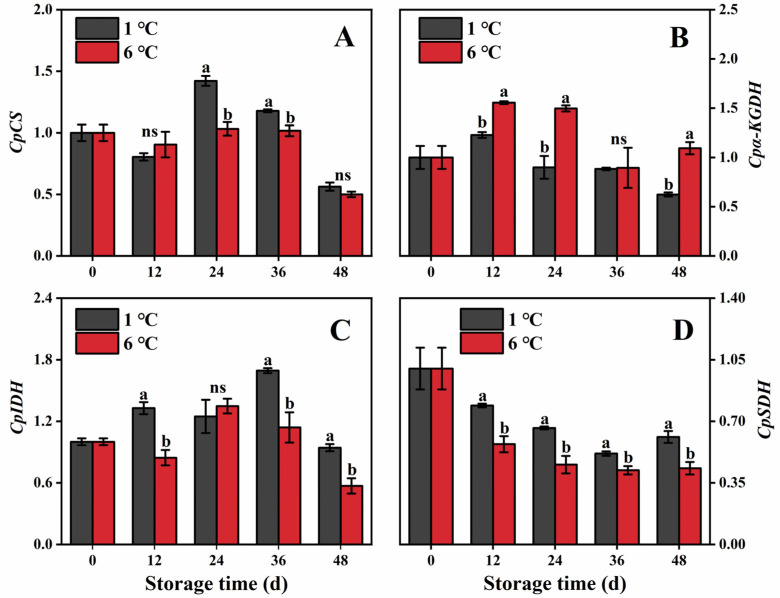
*CpCS* (**A**), *Cpα-KGDH* (**B**), *CpIDH* (**C**), and *CpSDH* (**D**) genes’ expression in papaya fruit during storage at 1 and 6 °C. Vertical bars represent the standard deviation of the means from three measurements. Different lowercase letters indicate significant differences (*p* < 0.05) between the 1 and 6 °C treatments in the same storage period. ns = Not significant.

During storage, the expression levels of the *CpCS* and *CpIDH* genes in papaya fruit initially increased, followed by a decline (Figure 7A,C). However, in fruit stored at 1 °C, there was an up-regulated expression of these two genes during a partial storage day. Nevertheless, this up-regulation was inhibited on days 12 and 24, respectively (Figure 7A,C). Similarly, the expression levels of the *Cpα-KGDH* gene in 1 °C- and 6 °C-stored fruit initially increased, peaked on day 12, and then decreased (Figure 7B). However, compared to 6 °C-stored fruit, the expression of *Cpα-KGDH* was suppressed in 1 °C-stored fruit during most of the storage period, except on day 36 (Figure 7B). On the other hand, fruit stored at 1 °C exhibited increased expression of *CpSDH* throughout the entire storage period, with the highest expression observed on day 24, which was 1.45 times that of 6 °C-stored fruit (Figure 7D).

### 2.7. Correlations between Indicators Were Analyzed

**Figure 8:**
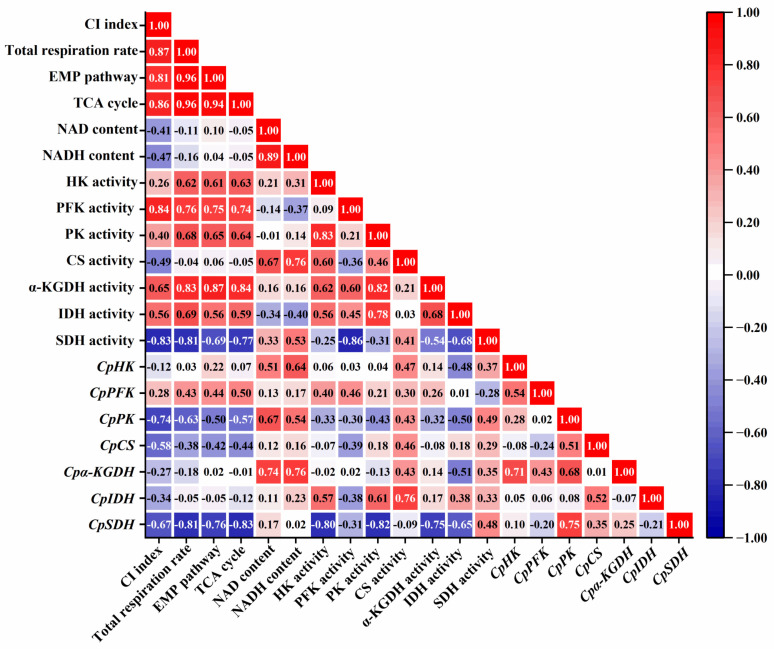
Correlations between indicators were analyzed. CI index: chilling injury index; Total respiration rate: the amount of oxygen absorbed or carbon dioxide released per unit time; EMP pathway: Embden–Meyerhof–Parnas pathway; TCA cycle: tricarboxylic acid cycle; NAD content: nicotinamide adenine dinucleotide content; NADH content: reduced nicotinamide adenine dinucleotide content; HK activity: hexokinase activity; PFK activity: phosphofructokinase activity; PK activity: pyruvate kinase activity; CS activity: citrate synthase activity; α-KGDH activity: α-ketoglutarate dehydrogenase activity; IDH activity: isocitrate dehydrogenase activity; SDH activity: succinate dehydrogenase activity; *CpHK*: the expression of hexokinase; *CpPFK*: the expression of phosphofructokinase; *CpFK*: the expression of pyruvate kinase; *CpCS*: the expression of citrate synthase; *Cpα-KGDH*: the expression of α-ketoglutarate dehydrogenase; *CpIDH*: the expression of isocitrate dehydrogenase; *CpSDH*: the expression of succinate dehydrogenase. Red coloration indicates positive correlations and blue coloration negative correlations. The intensity of the color is proportional to the degree of correlation.

Correlation analysis between the CI index and respiration pathway showed that there was a positive correlation between total respiration and CI (*r* = 0.87), EMP pathway and CI (*r* = 0.81), and TCA cycle and CI (*r* = 0.86) (Figure 8). Results of correlation analysis between components related to this pathway showed that HK (*r* = 0.61), PFK (*r* = 0.76), and PK (*r* = 0.65) were positively correlated with the EMP pathway. α-KGDH (*r* = 0.84) and IDH (*r* = 0.59) had a positive correlation with the TCA cycle, but SDH (*r* = −0.77) had a negative correlation, and CS (*r* = −0.05) was uncorrelated with the TCA cycle (Figure 8). Also, results showed that PFK (*r* = 0.46), CS (*r* = 0.46), IDH (*r* = 0.38), and SDH (*r* = 0.48) were in positive correlation with regulate their own genes, PK (*r* = −0.43) had a negative correlation with regulate their own genes, but HK (*r* = 0.06) and α-KGDH (*r* = 0.14) were uncorrelated with regulate their own genes (Figure 8). Furthermore, NAD (*r* = 0.1, *r* = −0.05) and NADH (*r* = 0.04, *r* = −0.05) were uncorrelated with EMP and TCA, but NAD and NADH had a positive correlation with CS (*r* = 0.67, *r* = 0.76), SDH (*r* = 0.33, *r* = 0.54), *CpHK* (*r* = 0.51, r = 0.64), *CpPK* (*r* = 0.67, *r* = 0.54), and *Cpα-KGDH* (*r* = 0.74, *r* = 0.76) (Figure 8).

## 3. Discussion

Respiration is essential in the postharvest physiological process of horticultural crops [30]. Postharvest fruit and vegetables exhibit a high respiratory rate and have an elevated proportion of the EMP-TCA pathway, leading to increased metabolic substrate consumption, which negatively affects their quality maintenance [22,28,31,32]. In our study, we observed that fruit stored at 1 °C exhibited a suppressed CI development phenomenon, which could be attributed to a lower respiratory rate of the EMP pathway and TCA cycle in the fruit during storage at 1 °C. This finding suggests that storing papaya fruit at 1 °C reduced the consumption of nutrient substances, which was confirmed by our previous report of Zhu et al. [33], in which higher contents of sucrose, fructose, and glucose in fruit during storage at 1 °C were described. Similar results were also observed in near-freezing-treated nectarines, indicating that high chilling tolerance might be correlated with low metabolism of the EMP pathway and a reduced rate of sucrose degradation [29].

NAD, acting as an electron acceptor, plays a role in accepting electrons to form NADH in the EMP pathway and TCA cycle [34,35]. Consequently, NADH content is used for assessing the respiratory intensity of the EMP-TCA pathway [5]. In comparison to papaya stored at 6 °C, storage at 1 °C resulted in a lower CI index and reduced levels of NADH. These findings further confirm that the mitigation of chilling injury in postharvest papaya during storage at 1 °C is related to a decrease in the activity of the EMP-TCA respiration pathway. Furthermore, a portion of NAD in the cytoplasm may transform into NADP via NAD kinase (NADK), promoting the generation of NADPH and enhancing stress resistance in plants [36,37]. In this work, the lower NAD content in fruit refrigerated at 1 °C suggests that more NAD in the cytoplasm could potentially be changed into NADP, leading to increased NADPH production through the PPP pathway. This, in turn, enhances the chilling tolerance of papaya fruit. Similarly, Li et al. [38] discovered that the exogenous application of epibrassinolide facilitated the change in the NAD(H) pool to NADP(H) by activating NADK, thereby promoting PPP activity and NADPH biosynthesis and enhancing chilling tolerance in postharvest bananas.

To elucidate the variation in the respiratory intensity of the EMP-TCA pathway in papaya fruit subjected to 1 and 6 °C, we investigated different enzymes related to respiration metabolism. In the EMP pathway, the phosphorylation of hexoses by HK plays a crucial role in signaling transduction. PFK converts fructose 6-phosphate to fructose-1,6-bisphosphate, and PK catalyzes the reversible conversion of phosphate and generates phosphoenolpyruvate [26,39]. Additionally, *CpHK*, *CpPFK*, and *CpPK* encoded the above enzyme, which regulated these enzyme functions generated. These genes also regulate the activity of corresponding enzymes in peony [40]. In our study, lower activities of HK, PFK, and PK were observed in papaya fruit stored at 1 °C. There was a positive correlation between the respiration rate of the EMP pathway and the activities of HK (*r =* 0.61) and PK (*r =* 0.65) (Figure 8). Moreover, 1 °C storage down-regulated the expression levels of *CpHK*, *CpPFK*, and *CpPK* genes more than 6 °C storage. The above results demonstrated that 1 °C storage could suppress CI development in papaya fruit, which is ascribed to inhibition of the EMP pathway at the transcript and enzyme levels. Similarly, Song et al. [18] found that NO-treated peaches decreased the EMP pathway by down-regulating the expression of PGI, PFK, and HK from the EMP pathway, which mitigated the occurrences of CI. It is worth noting that papaya stored at 1 °C exhibited higher activity of HK than that stored at 6 °C during the late period of storage, which may contribute to the hexose phosphorylation and the generation of sugar signal when papaya is subjected to chilling stress. Similar findings have also been reported in nectarine stored at 0 °C and 5 °C for five weeks [29].

CS, IDH, α-KGDH, and SDH are key enzymes in the TCA cycle [16]. The first crucial step in the TCA cycle is catalyzed by CS, which facilitates the irreversible conversion of acetyl-CoA oxaloacetate to citric acid [41]. Another pivotal enzyme in the TCA cycle is IDH, which acts as a rate-limiting enzyme and catalyzes the decarboxylation and dehydrogenation of isocitric acid to produce α-ketoglutaric acid [42]. Additionally, α-KGDH can catalyze the conversion of α-ketoglutaric acid to succinyl-coenzyme, which is accompanied by the reduction of NAD [43]. Additionally, among all enzymes, SDH is the only enzyme that is involved in both the TCA cycle and electron transfer chain. SDH can catalyze the oxidation of succinate to fumarate in the mitochondrial matrix to produce ATP [44]. These enzymes are encoded by *CpCS*, *CpIDH*, *Cpα-KGDH*, and *CpSDH*. Studies have shown that regulating the gene of IDH or α-KGDH in peony [40], the gene of SDH in apple [45], and the gene of CS in petunia [46] can affect the TCA cycle. In this study, we observed that storing the fruit at 1 °C led to a reduction in the activities of CS or α-KGDH while promoting IDH activity. Correlation analysis showed that α-KGDH activity (*r =* 0.84) and IDH activity (*r =* 0.59) were positively correlated with the respiration rate of the TCA cycle. Additionally, compared to 6 °C storage, 1 °C storage also inhibited the expression level of *Cpα-KGDH*. These findings collectively suggest that 1 °C storage can reduce the respiration rate of the TCA cycle by suppressing the activities of CS and α-KGDH but promoting IDH activity. Moreover, the SDH activity is a vital indicator to estimate the efficiency of the TCA cycle [28,44]. Notably, the papaya fruit stored at 1 °C exhibited higher activity and expression levels of SDH during storage than those papaya fruit stored at 6 °C. SDH is closely related to the production of energy and a higher energy level is conducive to the enhancement of chilling resistance in postharvest products [28]. Previous studies also found that higher energy status and an increase in SDH activity contributed to maintaining more energy and enhancing its resistance to the chilling of postharvest fruit [6].

## 4. Materials and Methods

### 4.1. Fruit Materials and Treatments

Papaya (*Carica papaya* L. cv. Daqing) fruits with a vibrant green color and approximately 10% yellow-green coloration were carefully handpicked from a papaya garden located in the Qiongshan district of Hainan Province, China. The papaya was transported to the laboratory within a 2 h timeframe. For the experiment, only fruit with consistent size, shape, and absence of any flaws or illness indications was chosen.

Initially, the fruit was sterilized for two minutes with a 1 g L^−1^ fungicide solution of prochloraz after being washed with water. The papaya was then separated into two groups at random, each group containing 180 fruits, and placed in individual polyethylene bags (40 cm × 30 cm, 0.01 mm thickness), including two fruits in each bag. Subsequently, the papaya underwent precooling overnight at 13 ± 1 °C before being transferred to refrigerators set at 1 °C and 6 °C, respectively, for a maximum duration of 48 days. Throughout the storage period, the relative humidity was maintained between 85% and 90%. At intervals of 11 days during storage, twelve fruits were sampled from each group. The fruit peels were rapidly frozen using liquid nitrogen, with a portion of the peel stored in a −40 °C refrigerator for subsequent determination of physiological indices and enzyme activity, and another portion of the peel was stored in a −80 °C refrigerator for the evaluation of gene expression, Additionally, ten fruits per replicate from each group were selected for assessing the CI index. A total of 60 fruits were randomly chosen from each treatment group to participate in the trials, which were performed in triplicate.

### 4.2. Evaluation of Chilling Injury (CI) Index

The main symptoms of papaya fruit chilling injury are pitting of the skin, scald, water soaking of the flesh, increased susceptibility to decay, etc. [7]. Among the symptoms, the pitting of the skin, scald, and decay were easy to see in the present research. Therefore, the chilling injury index was analyzed by these three symptoms’ area on the peel, following the method of Pan et al. [6]. The classification of CI area on the fruit surface, ranging from 0%, 0–10%, 10–30%, 30–50%, to >50%, was assigned respective grades of 0, 1, 2, 3, and 4. CI index was calculated by Σ (CI class × number of fruits in that class)/(the total number of fruits × 4).

### 4.3. Determination of Respiration Rate

The total respiration rate, Embden–Meyerhof–Parnas (EMP) pathway, and tricarboxylic acid (TCA) cycle were determined using a liquid-phase oxygen measurement system (Oxytherm + R, Hansatech Company, King’s Lynn, UK) following the methodology described by Tao et al. [20]. To perform the measurement, the peels (0.6 g) from the middle part of the fruits were finely cut into 1 mm × 1 mm pieces and placed in a 50 mL centrifuge tube and, subsequently, 50 mM potassium phosphate buffer solution (pH 7.4) was added to the tube and the sample was left to stand for 15 min in an incubator at 25 °C before measurement. The liquid-phase oxygen electrode was used to measure the respiration rate at a temperature of 25 ± 0.5 °C, with the rotor speed set at 1000× *g*. The total, EMP pathway, and TCA cycle respiration rates were reported in terms of μmol O_2_ kg^−1^ min^−1^.

### 4.4. Determination of NAD(H) Contents

The determination of NAD(H) contents was performed following a modified method based on Gibon et al. [47] and Mhamdi et al. [48]. To begin, 1 g frozen papaya peel was taken and mixed with 5 mL of either 0.1 M NaOH or HCI solution for NADH and NAD extraction, respectively. The resulting mixture was incubated for five minutes in boiling water. Subsequently, the supernatant was neutralized using 0.1 M NaOH or HCl, and the sample underwent a 10 min centrifugation process at 4 °C and 10,000× *g*. The obtained result was expressed as mmol kg^−1^.

### 4.5. Key Enzyme Activity in the EMP Pathway

#### 4.5.1. HK Activity

The determination of hexokinase (HK) activity was carried out with certain modifications based on the method described by Zhang et al. [40] and Wiese et al. [49]. To begin, papaya exocarp (1 g) was ground and homogenized in a cooled extraction buffer consisting of 50 mM Tris HCl (pH 8.5), 5 mM MgCl_2_·6H_2_O, 1 mM EDTA, 5 mM DTT, and 1% (*v*/*v*) Triton X-100. After centrifuging the homogenate at 13,000× *g* for 20 min at 4 °C, the supernatant was collected and used to make the crude enzyme extract.

For the HK assay, the supernatant (0.2 mL) was thoroughly mixed with 650 μL of Tris HCl (20 mM) containing 1.5 mM MgCl_2_·6H_2_O, 100 μL of 8 mM NADP-Na_2_, 100 μL of 20 mM ATP, 100 μL of 50 mM glucose, and 50 μL of 40 U mL^−1^ glucose-6-phosphate dehydrogenase. The absorbance at 340 nm was measured using a UV spectrophotometer (TU1901, Beijing General Analytical Instruments, Beijing, China). The HK activity was expressed as U kg^−1^ protein.

#### 4.5.2. PFK and PK Activities

Using assay kits purchased from Suzhou Keming Biotechnology Co., Ltd., Suzhou, China, the activities of PFK and PK were determined. The PFK and PK activities were quantified and expressed as U kg^−1^ protein.

### 4.6. Key Enzyme Activities in the TCA Cycle

#### 4.6.1. CS and α-KGDH Activities

To extract CS and α-KGDH, we mashed and homogenized 0.4 g of papaya exocarp in 1 mL of 0.01 M phosphate buffer (pH 7.4). Following that, the homogenate was centrifuged at 4500× *g* for 15 min at 4 °C. The resulting supernatant (50 μL) was utilized for measuring the activities of CS and α-KGDH using specific ELISA kits obtained from Jiangsu Meibiao Biotechnology Co., Ltd., Yancheng, China. The activity of CS and α-KGDH was expressed as U kg^−1^ protein.

#### 4.6.2. IDH and SDH Activities

Extraction of crude mitochondria from papaya exocarp was carried out following the method by Yang et al. [50], with a few adjustments. A total of 15 g of tissues was ground and homogenized using a cold extraction solution composed of 50 mM Tris HCl (pH 7.5), 1 mM EDTA, 0.25 M sucrose, 0.3 M mannitol, 1 g L^−1^ bovine serum albumin, 0.5% (*w*/*v*) polyvinylpyrrolidone, and 0.1% (*w*/*v*) cysteine. The homogenate was immediately filtered through a four-layer muslin cloth after 15 min of extraction and then centrifuged at 4500× *g* for 15 min at 4 °C. A second centrifugation was performed on the resultant supernatant at 4 °C and 16,000× *g* for 30 min. The resulting precipitate was resuspended in a buffer containing 10 mM Tris HCl (pH 7.5), 0.25 M sucrose, 0.3 M mannitol, and 1 mM EDTA. The mixture was stored at 4 °C for activity determinations of IDH and SDH.

The activity of IDH was determined using the improved method described by Keshavarzian et al. [51]. The 0.5 mL crude enzyme extract was a mixture containing 0.5 mL of 20 mM potassium phosphate buffer containing 1 mM MgCl_2_·6H_2_O, 1 mM NAD, and 10 mM DL-isocitric acid trisodium salt. The UV spectrophotometer was used to measure the change in absorbance at 340 nm. The IDH activity was expressed as U kg^−1^ protein.

The modified approach outlined by Yang et al. [50] was used to determine SDH activity. A total of 0.2 mL crude mitochondrial extract was mixed with 2 mL of 20 mM potassium phosphate buffer, 1 mL of 20 mM sodium succinate, and 0.1 mL of 1 mM di-p-chlorophenyl-methylcarbinol (DCPIP). Then, 0.1 mL of 10 mM methyl sulfenyl phenazine was used to start the reaction, and the UV spectrophotometer was used to measure the absorbance of 15 to 90 s at 600 nm. The SDH activity was shown as U kg^−1^ protein.

### 4.7. Gene Expression Analysis

Using real-time quantitative PCR, the expression levels of genes encoding key enzymes for the TCA cycle and EMP pathway were identified. The gene sequences of *CpHK* (GenBank Accession No. XM_022039184.1), *CpPFK* (XM_022052063.1), *CpPK* (XM_022052945.1), *CpCS* (XM_022031994.1), *CpIDH* (XM_022036344.1), *CpSDH* (XM_022051748.1), *Cpα-KGDH* (XM_022032975.1), and *CpActin* (FJ696416) in papaya fruit were obtained from NCBI. Primer design was conducted using Primer 6.0 software, and the details of all primers used are provided in Table 1. The 2^−ΔΔCT^ method reported by Livak and Schmittgen [52] was used to calculate gene expression levels.

### 4.8. Statistical Analysis

With three replicates for each treatment, randomized designs were used across all trials. An independent sample *t*-test was performed using SPSS 22.0 (IBM, Inc., Armonk, NY, USA) to analyze the data. Correlations between indicators were analyzed by Pearson’s correlation test. Graphs were generated using Origin 2022b software (Origin Lab Corp., Northampton, MA, USA). *p* < 0.05 was regarded as statistically significant, whereas *p* < 0.01 was regarded as very significant, indicating extremely significant differences between groups.

## 5. Conclusions

**Figure 9:**
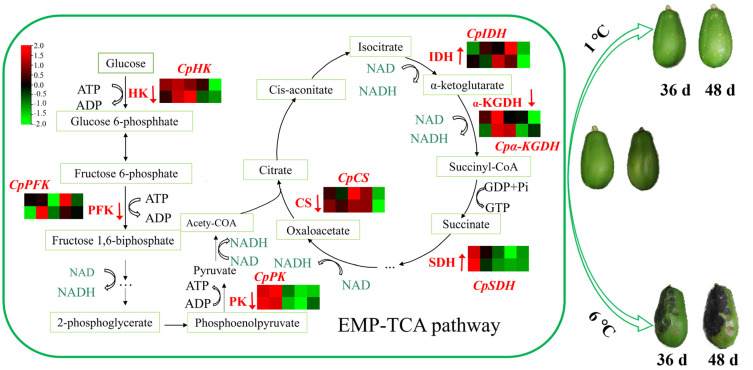
The probable mechanism of aberrant CI in papaya fruit at 1 °C and 6 °C. The red words and arrows in the figure represented enzymes that were up- and down-regulated when stored at 1 °C. The 6 °C-stored fruit showed the opposite result. Expression profiles of genes associated with the EMP-TCA pathway in papaya fruit during cold storage. The rows in each heat map represent the indicated genes, and the five columns indicate the 1 °C and 6 °C storage times (left to right): 0 d, 12 d, 24 d, 36 d, 48 d. The colors represent the differences in gene expression value between different samples in the heat map. Each value represents the mean for three replicates.

Storage of 1 °C reduced the activities of HK, PFK, PK, CS, and α-KGDH and regulated the expression of *CpHK*, *CpPFK*, *CpPK*, *CpCS*, and *Cpα-KGDH*, indicating that CI alleviation may be closely related to respiration suppression of the EMP-TCA pathway by the regulation of related enzymes and genes. The lower respiration rate of the EMP-TCA pathway contributed to the mitigation of substance consumption. Higher SDH activity was observed in papaya fruit stored at 1 °C, which ensures a sufficient supply of energy. Moreover, the lower NAD content in 1 °C-stored papaya fruit suggests that a portion of NAD is potentially utilized for the biosynthesis of NADPH in the PPP pathway. Consequently, postharvest papaya storage at 1 °C resulted in higher cold resistance compared to storage at 6 °C. The hypothetical model of this research is shown in Figure 9. This study deepens the understanding of CI and provides a theoretical basis for the development of methods for the alleviation of CI in tropical fruits.

## Figures and Tables

**Table 1:** Primers used for real-time quantitative PCR analysis.

Gene	Forward Primer (5′−3′)	Reverse Primer (5′−3′)	Accession Number
*CpHK*	TACAATTAGGTGGCAAGGAAGG	CGAATAATGCCTCAGAAGTTCC	XM_022039184.1
*CpPFK*	CCATAACCATTGCTTACTTCC	CATTGAGACCTAAGATGATTCC	XM_022052063.1
*CpPK*	CCCAATCCGTCGAGGTTATCTGTG	TCACTGCCGCCTTCAAATTCTCC	XM_022052945.1
*CpCS*	GACCGAAGCGAGATACTAC	AAGCCATTCCATTGTGACA	XM_022031994.1
*CpIDH*	TGTCTTGCTGTCCTCTGA	AATGCTGTTAGTGCTGGTT	XM_022036344.1
*CpSDH*	CCAGGAGGCTCTAGCAAATGTCTTC	TTCTCGCCGCTCAATCCAATGC	XM_022051748.1
*Cpα-KGDH*	AAGTCCTCTCCATCCTCAA	TGCCTTGCCAACATTCTT	XM_022032975.1
*CpActin*	TTAGCAACTGGGATGACATGG	TCGGTGAGAAGCACTGGGT	FJ696416

## Data Availability

The data presented in this study are available on request from the corresponding author.
